# Book Review: The Rationality Quotient—Toward a Test of Rational Thinking

**DOI:** 10.3389/fpsyg.2019.01889

**Published:** 2019-08-16

**Authors:** Mátyás Makay

**Affiliations:** Cognitive Psychology PhD Program, Department of Psychology, Eötvös Loránd University (ELTE), Budapest, Hungary

**Keywords:** cognitive bias, rationality, rationality quotient, rq, rationality test

Nobel Laureate American psychologist Daniel Kahneman (Nobel Memorial Prize in Economy Sciences in 2002), together with his colleague Amos Tversky, introduced the concept of cognitive biases, recurring, and systematic erroneous thinking patterns in our thinking, in the 1970's. Cognitive biases are oftentimes studied and researched in psychology but also in other fields of science. So much so American economist Richard Thaler was awarded another Nobel Memorial Prize in Economy Sciences (2017) for his contribution to behavioral economics, a new paradigm in Economics centered around the limited rationality of people.

It is quite remarkable that after countless important studies, decisive theoretical insights and a number of new scientific paradigms peaking in two Nobel prizes there were little success in developing a psychometric assessment device for rational thinking. “The Rationality Quotient—Toward a Test of Rational Thinking” by Professor Emeritus of Applied Psychology and Human Development at the University of Toronto Keith E. Stanovich and his colleagues make a strong point that rationality is indeed a measurable cognitive competence and can and shall be measured by quantitative assessment device. The book is the first comprehensive study in developing such a device and is to be considered a crucial square one for future efforts in this field.

The Rationality Quotient is a logical next step to Stanovich's previous books (Stanovich, 1999, 2009a,b, 2010), most notably to What Intelligence Tests Miss—The Psychology of Rational Thought of 2009 where the theoretical background of his concept of rationality was already elaborated in great detail. The Rationality Quotient builds heavily on that foundation and develops the theory into a practical assessment device.

Part I. provides a revised and concise version of the theory on rationality detailed in What Intelligence Tests Miss. Stanovich's concept is based on the Kahnemanian paradigm of dual process theory of thinking and it explains the roots of cognitive biases, thus the roots of dysrationalia (a term coined by Stanovich and referring to the inability to think and behave rationally despite having adequate intelligence), within this framework. It is important to note that Stanovich's approach is strongly normative as he sees cognitive biases as evident thinking errors which should be clearly avoided. While this is a highly logical and defendable standpoint it is far from being a universal view among cognitive psychologist.

A focal point of Stanovich's theory is the narrow concept of intelligence which confines the concept of intelligence to the set of mental abilities actually tested by existing IQ tests. There are quite relevant cognitive skills like sound judgment and decision making that are highly important to real-world behavior, affecting the way we plan, evaluate critical evidence, judge risks and probabilities, and make effective decisions. IQ tests fail to assess these skills of rational thought, even though they are measurable cognitive processes. What is more, argues Stanovich, intelligence and rationality show only small-to-medium correlation (Stanovich, 2009b), which makes an assessment device for rational thinking it even more relevant.

Part II. provides a detailed analysis of the components of the CART (Comprehensive Assessment of Rational Thinking) test including probabilistic and statistical reasoning, scientific reasoning, avoidance of miserly information processing, contaminated mindware, among others, and also different mental dispositions related to rationality. Many of the tasks are well-known and relatively simple questions from classic studies like Linda, the bank teller problem (designed by Kahneman and Tversky in 1983) or the Wason selection task (designed by Peter C. Wason in 1966) but there are some quite complex designs as well, like the Argument Evaluation Task. Based on the detailed guidelines for the design of the tasks and also for the suggested scoring system for every individual category of tasks one can rebuild the full RQ test (or a part of it) and start their own studies. Since the full test is quite sizeable and may take several hours to complete the authors designed two shorter versions of the test which are less comprehensive but whose results nevertheless correlate strongly with the full test.

Part III. offers a sizeable pool of psychometric data collected through the 3-year research that resulted in the “Rationality Quotient.” While this section is probably mainly interesting for specialized experts it also adequately demonstrates the solid empirical base built up through the research process. In the last two chapters the authors discuss the narrow and wider context of the CART, the remaining issues and also the social and practical implications of such a test.

Critics of intelligence tests, and to a lesser degree of the psychological construct of intelligence itself, argue that IQ tests minimize the importance of creativity, interpersonal skills, morality, empathy, and many other vital non-cognitive competence of a person. Stanovich et al. (2016) venture one step further when they argue, rather convincingly, that the IQ tests are incomplete assessment devices even in the cognitive domain since they miss a focal cognitive competence, namely the ability of rational thinking. In the Rationality Quotient they follow up this claim and provide the CART, an RQ test designed to measure rational thinking that is likely to become a standard psychometric tool in the future. They claim that the RQ is complementary to the IQ test and together provide a much wider assessment of one's cognitive capacity.

The Rationality Quotient is an effort of staggering scale and is an enormous accomplishment. The work is far from being completed, the authors themselves consider the RQ test a prototype and it certainly is. There are a number of open issues here including the missing but decisive biases like confirmation bias, myside bias, and bias blind spot. Also, some of the subtest of the cart, like Financial Literacy and Risk Knowledge are rather culture and sometimes country dependent. Wider issues like coachability and reusability of the test are still to be addressed.

It is clear that the Rationality Quotient is not intended to be the last word in the field of measuring rationality. Rather, it should be seen as a seminal and groundbreaking first step in measuring rationality a most important cognitive capacity. With its huge pool of data and its 62 page bibliography it is a solid starting point for further empirical and theoretical research.

## Author Contributions

The author confirms being the sole contributor of this work and has approved it for publication.

### Conflict of Interest Statement

The author declares that the research was conducted in the absence of any commercial or financial relationships that could be construed as a potential conflict of interest.
